# Morphological Brain Analysis Using Ultra Low‐Field MRI

**DOI:** 10.1002/hbm.70232

**Published:** 2025-06-30

**Authors:** Peter Hsu, Elisa Marchetto, Daniel K. Sodickson, Patricia M. Johnson, Jelle Veraart

**Affiliations:** ^1^ Bernard and Irene Schwartz Center for Biomedical Imaging, Department of Radiology New York University Grossman School of Medicine New York New York USA; ^2^ Vilcek Institute of Graduate Biomedical Sciences New York University Grossman School of Medicine New York New York USA; ^3^ Center for Advanced Imaging Innovation and Research (CAI^2^R), Department of Radiology New York University Grossman School of Medicine New York New York USA

## Abstract

Ultra low‐field (ULF) MRI is an accessible neuroimaging modality that can bridge healthcare disparities and advance population‐level brain health research. However, the inherently low signal‐to‐noise ratio of ULF‐MRI often necessitates reductions in spatial resolution and, combined with the field‐dependency of MRI contrast, challenges the accurate extraction of clinically relevant brain morphology. We evaluate the current state of ULF‐MRI brain volumetry utilizing techniques for enhancing spatial resolution and leveraging recent advancements in brain segmentation. This is based on the agreement between ULF and corresponding high‐field (HF) MRI brain volumes, and test–retest repeatability for multiple ULF scans. In this study, we find that accurate brain volumes can be measured from ULF‐MRIs when combining orthogonal imaging directions for T_2_‐weighted images to form a higher resolution image volume. We also demonstrate that not all orthogonal imaging directions contribute equally to volumetric accuracy and provide a recommended scan protocol given the constraints of the current technology.

## Graphical abstract

Accurate brain volume analysis from ultra low‐field (ULF) MRI is possible when leveraging complementary information from a series of images that have been acquired with high in‐plane spatial resolution relative to the slice thickness across multiple imaging directions. We further recommend a time‐efficient acquisition ordering for existing ULF scan protocols that prioritizes coronal T_2_‐weighted images to maximize morphological brain analysis.

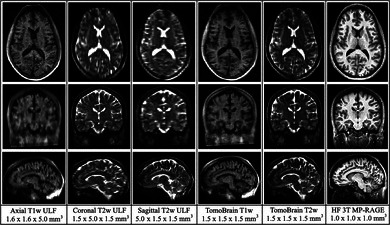

## 1 Introduction

To investigate the structure and function of the brain at the population level, across the lifespan, and in both health and disease, there is a need for quantitative biomarkers that can be extracted from accessible and scalable neuroimaging technology. Commonly used imaging biomarkers include morphological features such as gray or white matter volume (Giorgio and de Stefano 2013). Recent normative studies have shown that these biomarkers are sensitive to development, aging, and various psychiatric, neurological, and neurodegenerative disorders and diseases (Fox and Schott 2004; Fujita et al. 2023; Frenzel et al. 2020; Kalincik et al. 2012; Andravizou et al. 2019; Klok et al. 2019).

Magnetic resonance imaging (MRI), with a magnetic field strength of 1.5 T or higher, is the current standard for such brain volumetric analysis (Zhang et al. 2001; Friston et al. 2011; Fischl 2012). The development of MRI scanners with such high magnetic field strengths, collectively referred to as high‐field MRI (HF‐MRI), was promoted early on to maximize the signal‐to‐noise ratio (SNR) and spatial resolution of MRI data, while minimizing scan time (Webb 2016). However, there is a growing awareness that the push for strong magnetic fields comes at the expense of accessibility (Geethanath and Vaughan 2019; Fleming et al. 2021). Indeed, the accessibility of HF‐MRI remains low due to the specialized hardware, installation, and infrastructure requirements, and safety considerations associated with high‐magnetic‐field environments (Sarracanie et al. 2015; Ghadimi and Sapra 2025). Access is particularly limited in low‐ and middle‐income countries (LMICs), or rural and less populated areas within high‐income countries such as the USA (Hilabi et al. 2023; Jalloul et al. 2023; Murali et al. 2024; Burdorf 2021). This limited accessibility has exacerbated health care disparities and demographic biases in neuroimaging research studies and clinical trials (Quinn et al. 2021; DeBenedectis et al. 2022; van Dyck et al. 2023; Qin et al. 2022).

Ultra‐low field (ULF) MRI holds the promise of improving the accessibility of neuroimaging (Parasuram et al. 2023; Chetcuti et al. 2022; Liu et al. 2021; O'Reilly et al. 2021; Abate et al. 2024; Wu and Feng 2024; de Vos et al. 2021, 2023; Kuoy et al. 2022; Prabhat et al. 2021). While still in the early stages of development, ULF‐MRI is already making an impact in ambulatory settings and LMICs due to its (a) compact size and mobility, (b) reduced cost, (c) improved comfort, and (d) mitigated safety concerns (Kimberly et al. 2023; Mazurek et al. 2021; Guallart‐Naval et al. 2022; Deoni et al. 2022a; Altaf et al. 2023a, 2023b, 2024; Shen et al. 2024; Bauer et al. 2024; Beekman et al. 2022; Sheth et al. 2022; Turpin et al. 2020). ULF‐MRI devices operate at a magnetic field < 0.1 T, which is ~95% weaker than the current clinical state‐of‐the‐art. However, the inherently low SNR of ULF‐MRI often requires reductions in spatial resolution and, combined with the field‐dependent nature of MR contrast, poses challenges for both qualitative and quantitative analysis of ULF data (Webb and O'Reilly 2023; Marques et al. 2019; Arnold et al. 2023). For example, ULF images are, by default, not compatible with widely used brain segmentation tools such as FreeSurfer (Fischl 2012).

The intrinsic limitations of ULF‐MRI can be directly addressed by ongoing developments in deep learning (DL) methods for medical imaging (Lundervold and Lundervold 2019; Prince et al. 2020; Meng et al. 2023; Koonjoo et al. 2021; Zhao et al. 2024a, 2024b). Indeed, DL methods have proven to be effective in increasing SNR, improving spatial resolution, and performing contrast matching (Iglesias et al. 2021, 2023a; Lin et al. 2023; Lau et al. 2023; Man et al. 2023; Islam et al. 2023; Salehi et al. 2025). The adoption of such strategies for ULF‐MRI remains challenging due to the limited availability of imaging data for this emerging modality (Kimberly et al. 2023; de Leeuw Bouter et al. 2022). However, recently developed contrast‐agnostic image segmentation and analysis tools provide an avenue for brain morphometry from ULF‐MRI data (Briski et al. 2024; Cooper et al. 2024). In particular, *SynthSR* and *SynthSeg* can perform generative image enhancement and brain region segmentation, respectively, and are accessible through the widely adopted FreeSurfer toolbox (Iglesias et al. 2023a; Billot et al. 2023a).

In this exploratory study, we leverage these methodological advances to assess the accuracy and within‐subject repeatability of brain morphological feature extraction from ULF‐MRI data. We evaluate accuracy by measuring the agreement of brain tissue volumes, derived from subject‐matched ULF‐ and HF‐MRI data, respectively. We further measure within‐subject variability across repeated measurements and compare such variability across field strengths. Additionally, we perform an experimental design to maximize the efficiency of brain volume analysis, within the constraints of the current technology.

## 2 Methods

### 2.1 Data

All data were acquired with oversight by our institutional review board, with informed consent obtained from all participants. ULF‐MRI data were collected from 60 healthy control subjects (aged 22–75 years) on a Hyperfine Swoop scanner with a field strength of 64 mT (Parasuram et al. 2023; Chetcuti et al. 2022). All scans were performed using commercially available sequences provided by Hyperfine (software versions: 8.2.0–8.6.1). This includes a proprietary bias field correction and DL‐based reconstruction pipeline.

**FIGURE 1:**
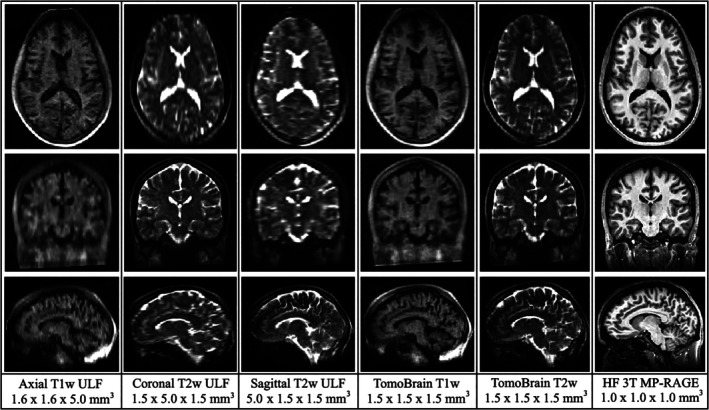
Image quality comparison between orthogonal ULF acquisitions (columns 1, 2, and 3), composite ULF *TomoBrain* images (columns 4 and 5), and a paired HF 3 T MP‐RAGE (column 6).

From each subject, ULF‐MRIs were collected with an 8‐channel head RF coil array using harmonized *T*_1_‐ and *T*_2_‐weighted (T1w and T2w) 3D fast spin‐echo (FSE) sequences in axial, coronal, and sagittal acquisition directions (Figure 1 and Table 1). The image resolution was 1.6 × 1.6 × 5.0 mm^3^ and 1.5 × 1.5 × 5.0 mm^3^ for the T1w and T2w sequences, respectively. The scan time for each individual image was approximately 6 min, resulting in a total scan time of approximately 36 min. Of our 60 subjects, 21 underwent a secondary *retest* ULF‐MRI scan on the same device, using the same imaging protocol.

**TABLE 1:** Overview of acquisition parameters for 3D ULF‐MRI data, including echo time (TE), repetition time (TR), inversion time (TI), echo train length (ETL), and scan time.

Contrast	B0 (mT)	TE (ms)	TR (ms)	TI (ms)	ETL	Voxel size (mm^3^)	Scan time (min:s)
T1w	0.64	6	1500	300	24	1.6 × 1.6 × 5.0	5:36
T2w	0.64	209	2000	—	80	1.5 × 1.5 × 5.0	5:46

In addition, the subject‐matched HF‐MRI data for 33 of the 60 subjects were retrospectively collected and analyzed. The HF‐MRI data were acquired at most 12 months before the ULF‐MRI scan. HF‐MRI scans were acquired at 1.5 T (*N* = 4), 3 T (*N* = 24), or 7 T (*N* = 5), all with T1w Magnetization‐Prepared Rapid Acquisition Gradient Echo (MP‐RAGE) contrast. In addition to these paired cases, 3 T data were collected from the Human Connectome Project (HCP; *N* = 45) public repository to benchmark test–retest performance of brain volumetric analyses for HF‐MRI (van Essen et al. 2013).

### 2.2 Image Preprocessing

All ULF‐MRI data underwent N4 bias field correction using advanced normalization tools (ANTs) (Isensee et al. 2019; Avants et al. 2014). We aligned all ULF‐MRI data using rigid registration to correct for subject motion between individual scans using the ANTs multivariate template construction script (Avants et al. 2011). The data from different fields‐of‐view (FoVs) (axial, coronal, and sagittal) were then combined by (a) regridding the individual images in a reference frame with an isotropic resolution of 1.5 × 1.5 × 1.5 mm^3^ and (b) averaging the regridded images. Both steps were performed using MRtrix3.0 tools (Tournier et al. 2019). Unless otherwise specified, the axial, coronal, and sagittal images were combined to create subject‐specific ULF‐MRI image volumes with an isotropic voxel size of 1.5 × 1.5 × 1.5 mm^3^. We will refer to the resulting images as T1w and T2w *TomoBrain*. A similar approach has previously been described by Deoni et al. (2022b).

Subject‐matched HF‐MRI data were also corrected for bias using the same ANTs functions before rigid registration of the images to the subject‐matched ULF‐MRI data. Images from public repositories had already undergone preprocessing, and additional processing was not performed. In an *ad hoc* analysis, we further downsampled our subject‐matched HF‐MRI data to 1.5 mm isotropic resolution before brain segmentation to evaluate the effect of resolution on volumetric agreement with ULF‐MRI (Supporting Information).

#### 2.2.1 Outlier Detection

During ULF image preprocessing, we performed a visual assessment of our data and determined that two cases were not sufficient for our analyses due to excessive motion and insufficient brain coverage (Figure S1). After removing these outliers, our final analysis consisted of 79 ULF images, including 33 subject‐matched ULF‐ and HF‐MRIs and 20 test–retest ULF‐MRIs.

### 2.3 Brain Segmentation

**FIGURE 2:**
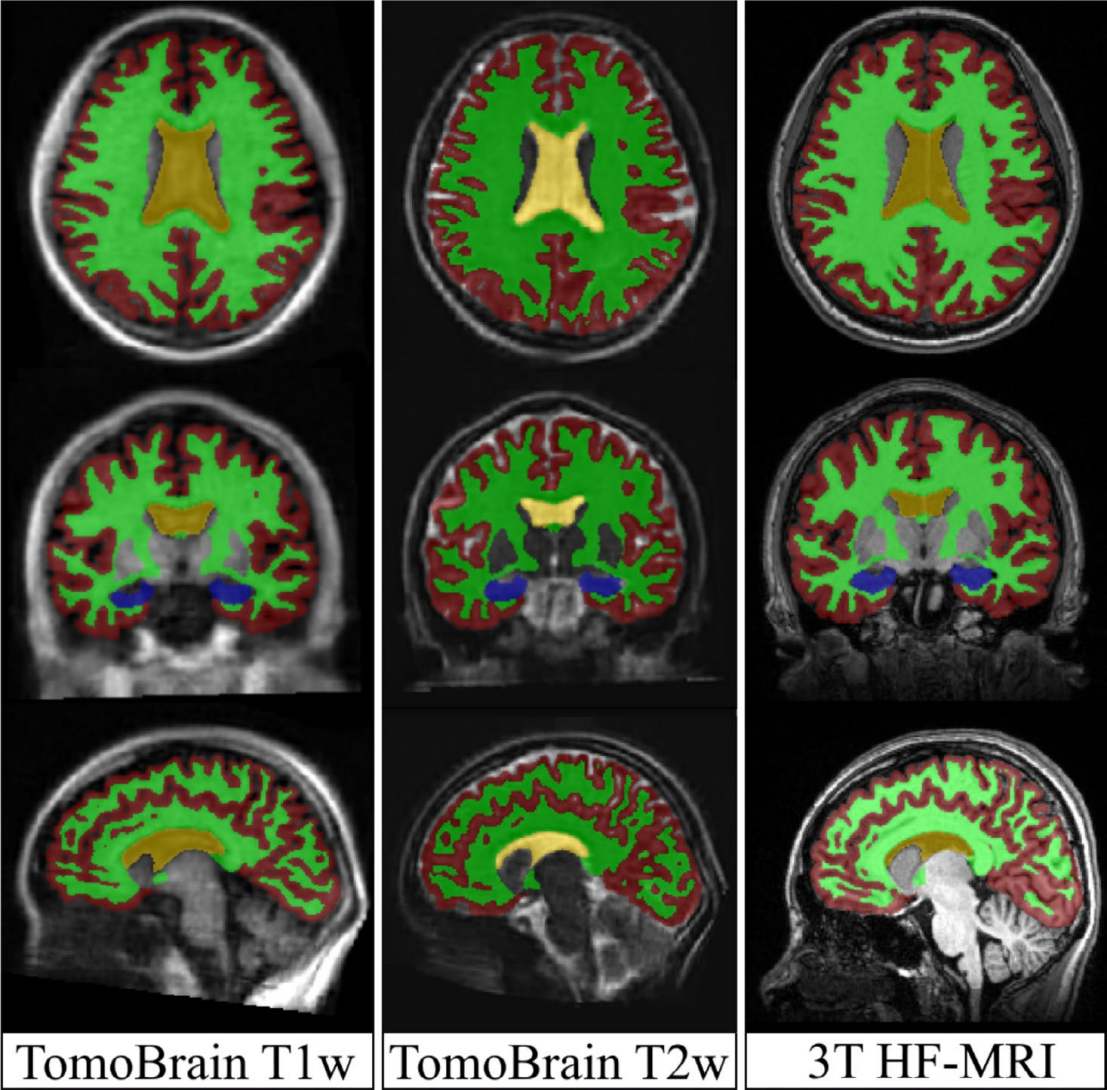
Example brain region segmentations made on the same subject on T1w *TomoBrain*, T2w *TomoBrain*, and 3 T HF‐MRI for gray matter (red), white matter (green), hippocampus (blue), and lateral ventricles (yellow).

Automated segmentation was performed using SynthSeg+, a contrast‐agnostic brain segmentation tool within the FreeSurfer software library (version 7.4.1) (Billot et al. 2023b). We extracted the volumes of total cerebral gray matter (GM), total cerebral white matter (WM), hippocampus (HC), and lateral ventricles (LV), as well as the total intracranial volume (ICV) (Figure 2). All nominal values were tabulated per subject and per available image contrast.

### 2.4 Statistical Analyses

#### 2.4.1 Intra‐Subject Correlation Across Field Strengths

We quantified the linear correlation between the brain morphological features from subject‐matched ULF‐ and HF‐MRIs (*N* = 33 paired cases) using the Pearson Correlation Coefficient (ρ). The analysis was performed independently for T1w and T2w *TomoBrain* data.

#### 2.4.2 Intra‐Subject Agreement Across Field Strengths

We evaluated the agreement of brain morphological features between subject‐matched ULF‐ and HF‐MRI data using Lin's Concordance Correlation Coefficient (CCC) and the Dice Similarity Coefficient (DSC) (Akoglu 2018; Zou et al. 2004). Lin's CCC quantifies the conformity of bivariate pairs of observations relative to one another in terms of accuracy and precision. In its essence, the CCC is a product of a coefficient of accuracy ($C_{a}$), that measures systematic offsets, and the Pearson correlation coefficient (ρ) (Lin 1989). This takes the following form:

(1) $$\mathrm{CCC}=\rho C_{a}=\frac{2\rho \sigma_{x}\sigma_{y}}{\sigma_{x}^{2}+\sigma_{y}^{2}+{\left(\mu_{x}-\mu_{y}\right)}^{2}}$$

where σ_x_ and σ_y_ denote the standard deviation, *σ*_*x*_^2^ and *σ*_*y*_^2^ represent covariance, and *μ*_*x*_ and *μ*_*y*_ are the means for our ULF‐ and HF‐MRI cohorts, respectively. Hence, Lin's CCC supplements ρ by quantifying systematic biases between the agreement of paired observations. Our interpretation of Lin's CCC follows the guidelines of Koo and Li (2016): > 0.9: excellent, 0.75–0.9: good, 0.5–0.75: moderate, and < 0.5: poor.

We also analyzed the shape of segmentations made across field strengths using the DSC. This metric measures spatial overlap between brain segmentations of subject‐matched ULF‐ and HF MRI data. Before segmentation, *TomoBrain* images and HF‐MRIs were resampled to 1 mm isotropic voxel size and aligned using ANTs rigid registration (Avants et al. 2014). This specific image alignment was not required and not performed for the volumetric comparison using the CCC.

#### 2.4.3 Within‐Subject Repeatability

We quantified test–retest variability (*N* = 20 test–retest cases) using two metrics for repeatability (Akoglu 2018). For our purposes, repeatability refers to the consistency of a measurement when repeated over time using the same imaging hardware. First, we computed the coefficient of variation (CoV) across repeated measurements by using the scaled mean absolute difference as a proxy for the standard deviation (Veraart 2021). More specifically, we computed

(2) $$\mathrm{CoV}=\frac{\sqrt{\pi }}{N}\sum_{i=1}^{N}\frac{\left|a_{i,1}-b_{i,1}\right|}{a_{i,1}+b_{i,1}}$$

to measure the variation between subjects across repeat measurements (Koo and Li 2016). Here, *N* refers to the total number of cases, and *a*_*i*,*1*_ and *b*_*i*,*2*_ denote the *i*th case for the first and second time point data, respectively. Second, we measured Lin's CCC to quantify the agreement between repeat measurements within subjects, and to quantify systematic biases in our data. This utilizes Equation (1), substituting the first and second time point data for *x* and *y*, respectively. Both metrics were also computed for the HCP test–retest dataset as a reference standard for HF‐MRI. We then visualized the differences between ULF and HCP HF test–retest variability using a Bland–Altman plot with 95% confidence intervals for each volume of interest. We additionally report the repeatability of segmentation shape metrics based on the mean DSC across our test–retest data.

#### 2.4.4 Age–Volume Interactions

We explored the sensitivity of ULF‐MRI to age‐related morphological changes across the lifespan. Using linear regression, we measured (a) the age‐dependency of the difference between subject‐matched ULF‐ and HF‐MRI data (*N* = 33) and (b) the age‐dependency of normalized brain volumes, as derived from our ULF‐ (*N* = 60, single scan) and HF‐MRI data (*N* = 33). For each regression, we computed the slope, alongside its uncertainty. To mitigate sampling biases within our cohort, we utilized bootstrapping in four age ranges (22–34, 35–47, 48–60, and 60+) to calculate age–volume trends per brain region of interest. We then sampled 5 subjects with replacement per age range and calculated the age–volume trend for 500 trials with 95% confidence (Davison and Hinkley 1997).

#### 2.4.5 Recommended Acquisition Ordering

All analyses were initially performed with *TomoBrain* images that were constructed by merging data from three orthogonal views. We additionally tested the performance of ULF‐MRI brain volume analysis when using subsets of the imaging data to design a more time‐efficient protocol. Performance was evaluated using brain segmentations of GM, WM, HC, and LV with Lin's CCC between ULF‐ and paired HF‐MRI data (*N* = 33).

## 3 Results

### 3.1 Intra‐Subject Correlation Across Field Strengths

**FIGURE 3:**
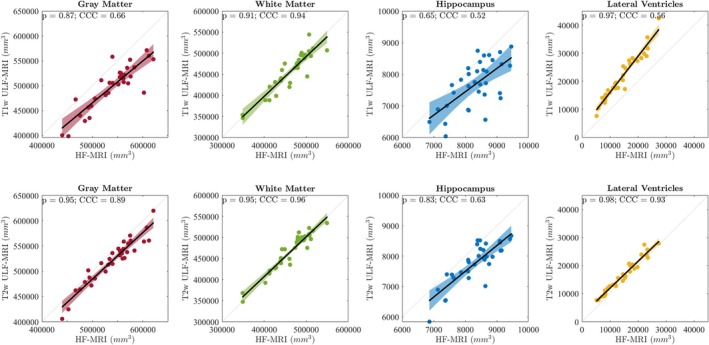
Bivariate scatter plots comparing four estimated brain volumes (GM, WM, HC, and LV) for paired ULF‐MRI (*y*‐axis) and HF‐MRI (*x*‐axis). Row 1 showcases T1w ULF‐MRI, and row 2 showcases T2w ULF‐MRI. Pearson's correlation coefficient and Lin's CCC metrics are displayed on each plot.

The bivariate scatter plots for each of the volumetric features from ULF‐ and HF‐MRI are displayed graphically in Figure 3. We observe a strong linear correlation between volumetric features estimated from T2w ULF‐ and HF‐MRI. The Pearson correlation coefficient (ρ) was measured for each brain region, with values of 0.83, 0.95, 0.95, and 0.98 for HC, GM, WM, and LV, respectively. The same brain volume features exhibit reduced correlations when derived from T1w ULF images, particularly for HC. Here, ρ was measured to be 0.65, 0.87, 0.91, and 0.97 for HC, GM, WM, and LV, respectively.

### 3.2 Intra‐Subject Agreement Across Field Strengths

Alongside the Pearson correlation in Figure 3, we also calculated systematic offsets of brain volumes between ULF‐ and HF‐MRI with Lin's CCC. We observe significant deviations from the unit line for all metrics, particularly for GM and HC, indicating significant differences in brain volume measurements between scanners. We observe that brain morphological features derived from T2w ULF‐MRI are more consistent with subject‐matched HF‐MRI, compared to T1w ULF‐MRI. Overall, the agreement for T2w ULF‐MRI (CCC = 0.96, 0.93, 0.89, and 0.63, for WM, LV, GM, and HC, respectively) is *excellent* for WM and LV, *good* for GM, and *moderate* for HC. A summary of these metrics is presented in Table 2.

**TABLE 2:** Summary of volumetric agreement metrics between ULF‐MRI and paired HF‐MRI for *TomoBrain* T1 and *TomoBrain* T2.

	GM			WM			HC			LV		
	*p*	CCC	DSC	*p*	CCC	DSC	*p*	CCC	DSC	*p*	CCC	DSC
TomoBrain T1	0.87	0.66	0.71	0.91	0.94	0.81	0.65	0.52	0.78	0.97	0.56	0.75
TomoBrain T2	0.95	0.89	0.74	0.95	0.96	0.82	0.83	0.63	0.82	0.98	0.93	0.82

*Note:* This includes the Pearson correlation coefficient (p), Lin's concordance correlation coefficient (CCC), and the mean dice similarity coefficient (DSC) calculated across subjects for the GM, WM, HC, and LV.

The shape of segmentations made on T2w ULF‐MRIs was more consistent with that of the paired HF‐MRIs based on the mean DSC across subjects, with values of 0.74 ± 0.02 for GM, 0.82 ± 0.02 for WM, 0.82 ± 0.05 for HC, and 0.82 ± 0.07 for LV. In comparison, T1w ULF‐MRI had lower DSC values, with 0.71 ± 0.04 for GM, 0.81 ± 0.03 for GM, 0.78 ± 0.06 for HC, and 0.75 ± 0.07 for LV. Overall, this indicates better overlap between the segmented brain regions of T2w ULF‐MRI and HF‐MRI.

### 3.3 Within‐Subject Repeatability

**FIGURE 4:**
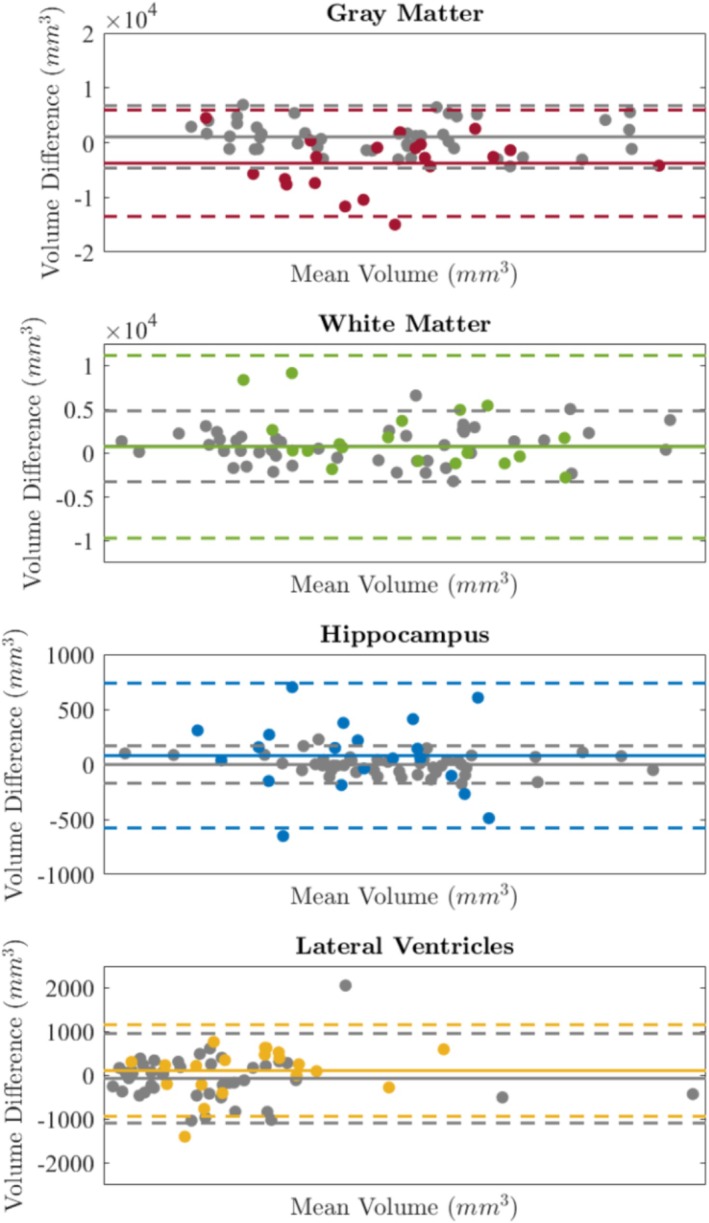
Bland–Altman plot comparing test–retest repeatability of T2w *TomoBrain* ULF‐MRI (color) and HCP HF‐MRI (gray scale) per brain volume.

In Table 3, we summarize the test–retest analysis of repeat scans, measured using CCC and CoV. In Figure 4, the test–retest repeatability is visualized using Bland–Altman plots. For T2w ULF data, the CoV varies from 0.60 to 3.04, whereas for T1w ULF data, the CoV is larger overall, with values ranging between 1.86 and 4.44. When compared to HF‐MRI, the CoV is significantly higher for GM, WM, and particularly HC, whereas it is similar across field strengths for LV. When quantified using Lin's CCC, within‐subject repeatability is *good* to *excellent* when using T2w ULF‐MRI data.

**TABLE 3:** Test–retest comparisons of ULF *TomoBrain* T1w, ULF *TomoBrain* T2w, and HCP 3 T reference data across ICV, GM, WM, HC, and LV volumes.

	ICV			GM			WM			HC			LV		
	CCC	CoV	DSC	CCC	CoV	DSC	CCC	CoV	DSC	CCC	CoV	DSC	CCC	CoV	DSC
HCP 3 T	1.00	0.26	0.99	1.00	0.41	0.87	1.00	0.34	0.90	0.99	0.74	0.89	1.00	2.76	0.88
TomoBrain T1	0.93	1.88	0.98	0.94	2.03	0.84	0.96	1.86	0.90	0.68	4.44	0.85	0.99	3.62	0.92
TomoBrain T2	0.97	1.17	0.98	0.99	0.79	0.85	0.99	0.60	0.90	0.97	3.04	0.88	0.99	2.97	0.93

*Note:* Metrics include Lin's CCC, the CoV in terms of percentage, and the mean DSC between segmentations across subjects.

T2w ULF‐MRI had higher mean DSC for all segmentations between test and retest scans (0.98 ± 0.01 for ICV, 0.85 ± 0.04 for GM, 0.90 ± 0.02 for WM, 0.88 ± 0.02 for HC, and 0.93 ± 0.03 for LV), compared to T1w ULF‐MRI (0.98 ± 0.01 for ICV, 0.84 ± 0.06 for GM, 0.90 ± 0.03 for WM, 0.85 ± 0.05 for HC, and 0.92 ± 0.03 for LV). For reference, the mean DSC values for the HCP reference data were 0.99 ± 0.01 for ICV, 0.87 ± 0.09 for GM, 0.90 ± 0.08 for WM, 0.89 ± 0.1 for HC, and 0.88 ± 0.1 for LV.

### 3.4 Age–Volume Interactions

**FIGURE 5:**
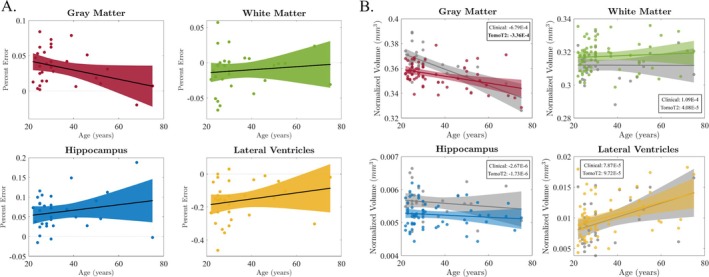
(A) Percent error between T2w ULF *TomoBrain* and paired HF‐MRI data (*y*‐axis) as a function of age (*x*‐axis). Trends are visualized through linear regression, but none reach statistical significance. (B) Age–volume trends for T2w ULF *TomoBrain* (colored) and HF‐MRI data (gray scale) comparing brain volumes of interest, normalized by total intracranial volume (*y*‐axis) and age in years (*x*‐axis). The annualized atrophy rates for each volume were computed from our bootstrapped analysis and are shown in each plot for the Clinical HF‐MRI and T2w *TomoBrain* cohorts. Statistical significance is denoted in bold at 95% confidence.

Figure 5A showcases the percent error between brain volumes of ULF T2w *TomoBrain* and subject‐matched HF‐MRI, plotted for increasing age across our cohort of images. None of these trends was statistically significant at the 95% confidence level. Figure 5B presents the age‐volume trends for the same brain regions, extending the comparison across our full set of ULF and HF images. This also displays the annualized atrophy rates of normalized volumes calculated from our bootstrapped analysis. We observe one statistically significant difference when comparing the ULF GM trend to that of the HF trend at 95% confidence. We also note that none of the bootstrapped atrophy rates significantly differed from the non‐bootstrapped rates (data not shown).

### 3.5 Recommended Acquisition Ordering

**FIGURE 6:**
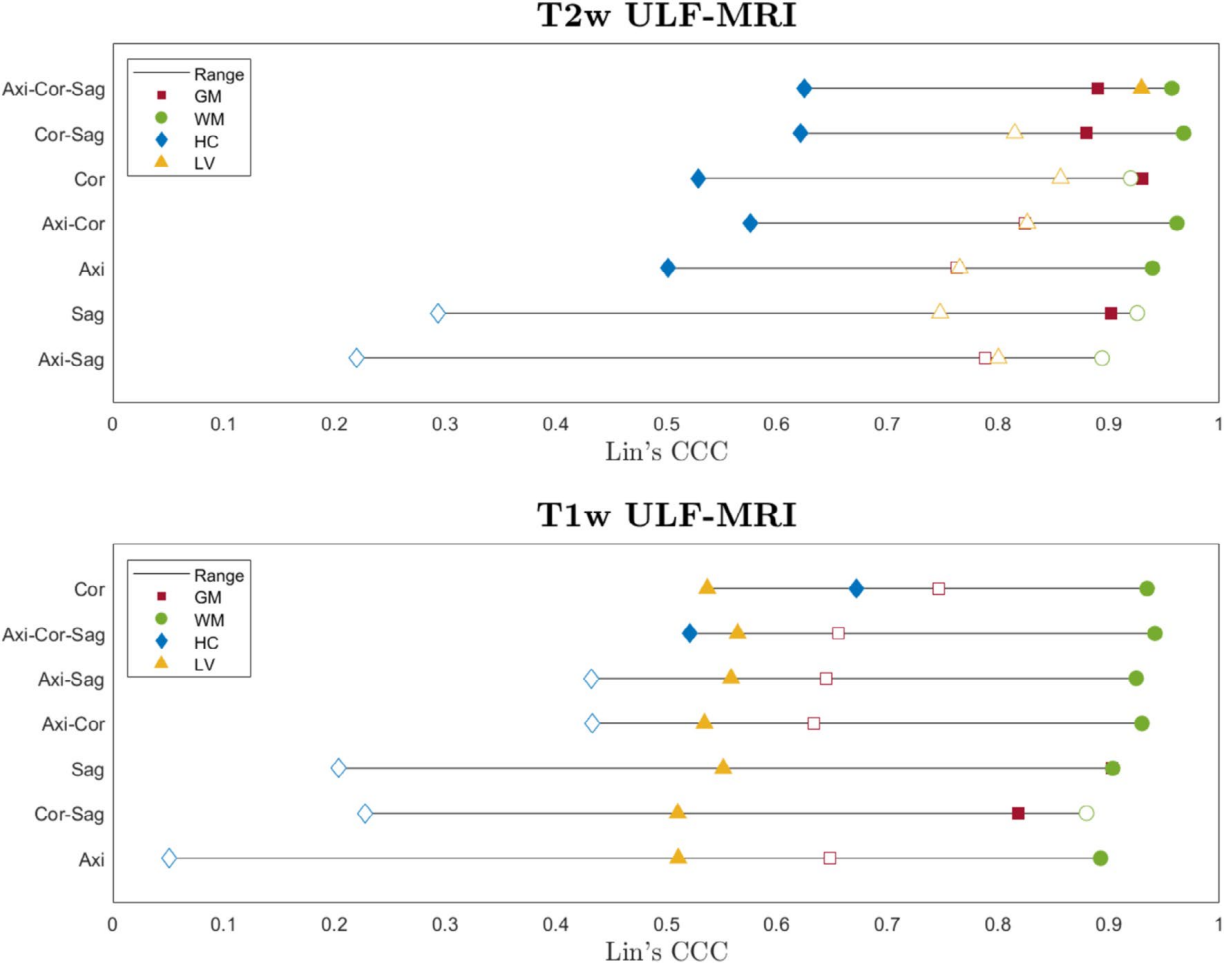
Recommended acquisition ordering comparison for different ULF‐MRI acquisition combinations per contrast, including single acquisitions, pairs of acquisitions, and full acquisition TomoBrain. The *y*‐axis lists the acquisition combinations, while the *x*‐axis shows Lin's CCC with paired HF‐MRI data for each volume of interest (represented by colored shapes). Unfilled shapes indicate significantly worse performance for that brain region compared to the best result for that contrast, based on the 95% confidence interval. Each plot is arranged from top to bottom according to overall performance across all volumes.

We evaluated intra‐subject agreement across all individual, partial, and full combinations of T1w and T2w ULF‐MRI acquisition directions to determine the most efficient configuration of ULF images, specific to our scan protocol, for accurate extraction of brain region volumes based on scan time using a ranked ordering in Figure 6. The combination of three T2w ULF acquisitions, that is, the *TomoBrain*, provides the highest overall performance, determined by Lin's CCC with paired HF‐MRI data (CCC = 0.8902, 0.9570, 0.6252, and 0.9296 for GM, WM, HC, and LV, respectively), when compared to any combination of T1w data or subsets of T2w data. For combinations of two T2w ULF acquisitions, the coronal–sagittal (Cor–Sag) does not significantly reduce the performance of GM, WM, and HC compared to the T2w *TomoBrain*. We also observe that the T2w coronal provides the highest agreement for an individual image acquisition compared to the axial or sagittal directions. The full comparisons/results are presented in Table S1.

## 4 Discussion

In this study, we have assessed the feasibility of ULF‐MRI for brain volume analyses. We performed a comparison of subject‐matched brain volume measurements (GM, WM, HC, and LV) across field strengths and then evaluated the consistency of brain structures over repeated measurements. In summary, we observed (a) strong correlations between brain morphological features that were derived from ULF‐MRI and paired HF‐MRI, respectively, (b) *good* to *excellent* within‐subject repeatability between multiple ULF scans, and (c) discrepancies in the nominal values of estimated GM, HC, and LV volumes across field strengths.

The strong correlations (ρ = 0.95, 0.95, 0.83, and 0.98 for GM, WM, HC, and LV, respectively) between T2w *TomoBrain* ULF‐ and paired HF‐MRI highlight the feasibility of extracting clinically relevant information, such as brain tissue volumes, from a more accessible neuroimaging modality. Overall, these results reproduce the findings of Deoni et al. (2021) and Cooper et al. (2024), but further extend their generalizability from children and young adults to an aging population. Brain volumetrics are relevant biomarkers not only for the diagnosis of neurodegenerative diseases, but also for the study of brain aging at the population level. Indeed, there is growing interest in harmonizing such MRI‐derived biomarkers across studies to develop a population‐representative statistical model, *brain charts* of the aging brain (Bethlehem et al. 2022). The ability to extract these biomarkers using more accessible, possibly more mobile, neuroimaging technology is critical to address health care disparities and mitigate known biases in study cohorts (Betancourt et al. 2019; Lawson et al. 2022).

Despite the strong correlations, there are systematic differences in the estimated GM, HC, and LV volumes when comparing ULF‐ to HF‐MRI. Indeed, the GM and HC volumes are underestimated (mean percentage error of about 3% and 6%, respectively), while an overestimation of the LV (approximately 16%) is observed. In general, it is known that MRI‐derived brain volume measures are often not robust to variations in vendor, scanner, or software, even when the field strength is constant (Jovicich et al. 2009). In fact, there is an emergence of data harmonization tools to mitigate such site effects to pool data across studies (Pomponio et al. 2020; Liu and Yap 2024). While some discrepancies in brain tissue volume may be decreased by adopting such strategies, we must understand their cause and reduce them as much as possible. We identify three potential sources of tissue volume discrepancy across field strengths: spatial resolution, field‐dependency of image contrast, and spatial deformations.

- *Spatial resolution*: Spatial resolution is known to have an impact on brain segmentation (Van Leemput et al. 2003; Henschel et al. 2022). In addition to our primary analysis, we downsampled our HF‐MRI data to match the ULF *TomoBrain* voxel size prior to brain segmentation. When evaluating the volumetric agreement between ULF‐MRI and resolution‐matched HF‐MRI, we observed noticeable increases in the agreement of brain volumes across field strengths when compared to our reported results (Figure S2). For example, the CCC of the GM increased from 0.89 to 0.94, and the HC increased from 0.63 to 0.72.
- *Field dependency of image contrast*: One limitation of the study is that brain tissue volumes of HF‐MRI are considered the *ground truth* values. However, tissue boundary definitions can be field‐dependent due to interactions between tissue microstructure and field‐dependent MR relaxation times (Natu et al. 2019). For example, it has been previously demonstrated that the GM/WM boundary, as apparent on MRI, is associated with myelination (Mezer et al. 2013). Mediated by the impact of microstructure on relaxation times, such microstructural changes have manifested themselves as apparent changes in cortical morphology in a developmental cohort. The field dependency of relaxation times, and their interaction with microstructure, might shift the GM/WM boundary across field strengths. The magnitude of this effect could also be age‐specific, given that myelination levels vary across the lifespan (Yeatman et al. 2014). We hypothesize that this might further explain our preliminary observation of reduced atrophy rates in normalized GM volume derived from ULF‐MRI compared to subject‐matched HF‐MRI. However, larger samples with more uniform age binning and an evaluation of myelin‐sensitive markers will be needed to further test this hypothesis.
- *Spatial Deformations*: Permanent magnets have relatively low magnetic field homogeneity compared to clinical systems (Ren et al. 2019; Manso Jimeno et al. 2023). Such inhomogeneities can cause anatomical deformation, potentially impacting the nominal size and shape of tissue types (Cooley et al. 2021). These distortions may impede the measurement of voxel‐by‐voxel segmentation metrics, such as the dice similarity coefficient (Zou et al. 2004). However, their impact will vary across scanner designs and image processing pipelines to the extent that they mitigate or correct for such inhomogeneities (O'Reilly et al. 2021; Block et al. Early View).

Downsampling HF‐MRI data prior to brain segmentation results in better correspondence with our ULF‐MRI data, highlighting the impact of spatial resolution on brain segmentation and volumetric analyses. Hence, we hypothesize that increasing the spatial resolution of ULF‐MRI is a critical direction in support of such analyses. In this study, we adopted the idea of *TomoBrain* to improve the spatial resolution of ULF‐MRI by combining a series of images with anisotropic voxel sizes that are acquired along orthogonal imaging directions (axial, coronal, sagittal) to form a proxy image with an isotropic voxel size of 1.5 × 1.5 × 1.5 mm^3^. However, merging such ULF‐MRI data still misses the *corners* in the detail‐encoding regions of the combined *k*‐space. To address this, we explored 3D total generalized variation (TGV) to fill these outer *k*‐space corners, but did not find statistically different results compared to the original images (Knoll et al. 2011). In follow‐up research, we will focus our efforts on the development of AI technology for this purpose.

The *TomoBrain* approach is a practical solution to increase ULF spatial resolution, given the constraints of the current imaging hardware and software. While the resolution is fixed, rotating FoVs can fill gaps in *k*‐space to create a higher resolution ULF image with improved SNR. However, this solution is not very time‐efficient and is prone to subject motion (Deoni et al. 2021, 2022b). In fact, we observed that some instances of merging data lowered image quality and subsequent volumetric performance (Figure 6). Therefore, we performed an analysis to find a recommended acquisition order for such imaging series, highlighting that T2w images are generally more attuned to volumetric analysis and the coronal acquisition is a key imaging direction. We find that GM, WM, and HC volumes can be estimated without a significant performance loss compared to *TomoBrain* using only T2w coronal and sagittal directions (Cor‐Sag), resulting in an imaging protocol of approximately 12 min. Overall, our results indicate that T2w ULF images should be prioritized for volumetric analysis, supporting previous findings of Deoni et al. (2021). However, in contrast to previous efforts (Deoni et al. 2021; Iglesias et al. 2023b), we recommend acquiring coronal images first to maximize the efficiency of brain volume analysis, in case data acquisition gets interrupted, followed by sagittal and then axial.

Compared to previous studies, such as Deoni et al. (2021) and Cooper et al. (2024), we present test–retest data to quantify the precision of the proposed methods. Although segmentation overlap was similar for test–retest cases across field strengths, we demonstrated that ULF‐MRI brain volume analysis suffers from reduced precision compared to HF‐MRI due to reduced spatial resolution, reduced signal‐to‐noise ratio, and possible subject motion. While the test–retest repeatability for the LV volume is comparable across field strengths, we observe an increased CoV for GM, WM, and HC. For GM and WM, the CoV is increased by approximately twofold, while for the HC, the loss in precision is more pronounced when compared to HF‐MRI experiments. In cohort studies, any loss in the precision of a metric can be compensated by an increased sample size (Serdar et al. 2021; Althubaiti 2022). We hypothesize that the increased accessibility and scalability of ULF‐MRI, compared to HF‐MRI, may compensate for the observed loss of precision by facilitating large‐scale studies. Our follow‐up work will explore the potential to further improve the precision of brain volume measurements from ULF‐MRI data after enhancing SNR and spatial resolution using DL methods.

The development and validation of ULF‐MRI acquisitions, processing, and analysis approaches rely on extensive subject‐matched data across field strengths. The acquisition of such datasets is time‐consuming, potentially delaying scientific progress. To accelerate comparative studies and to minimize the burden on research participants, we opted to include previously acquired HF‐MRI data of research participants for a secondary analysis in our study. As a result, our HF‐MRI data includes some heterogeneity in scan parameters, particularly in field strength (1.5, 3, and 7 T). However, we verified that this variability does not impact the findings of this study. Specifically, when restricting the analysis to 3 T scans only, we found no statistically significant differences in the volumetric analyses.

Alongside imaging sequences, we also had to adopt ULF‐MRI reconstruction pipelines, which differ from conventional HF‐MRI to address specific challenges, such as low SNR. In particular, proprietary DL reconstruction and bias correction are performed as part of default acquisition pipelines. This means our results, especially the quantitative evaluation metrics, are subject to change as such software is updated. However, the same applies to DL‐based image segmentation tools that remain under active development. Follow‐up research might include the development of unified pipelines, going from ULF *k*‐space data to both an enhanced image and a corresponding segmentation.

Within the scope of this work, DL technology is already leveraged for automated brain segmentation. We here use SynthSeg+ as an existing contrast‐agnostic solution that is able to segment ULF‐MRI (Billot et al. 2023b). However, current solutions, such as SynthSR and SynthSeg, rely on synthetic training data that emulates ULF features to learn corresponding image enhancement and segmentation, leaving them prone to “domain shift” errors (Iglesias et al. 2023a; Billot et al. 2023a; Chen et al. 2021; Kondrateva et al. 2021). While such strategies may be sufficient for brain morphology measurements in normative studies, their clinical use may ultimately be limited if pathology is not simulated adequately (Bhadra et al. 2021; Gottschling et al. 2020). We believe these concerns can be mitigated by training and validating such models with real, subject‐matched ULF‐ and HF‐MRI data, incorporating a diverse set of healthy controls and patient scans across the lifespan.

Within the current workflow, from image reconstruction to analysis, DL technology is advancing the utility of ULF‐MRI for clinical and research applications (Chetcuti et al. 2022; Mazurek et al. 2021; Billot et al. 2023b). While we identify targets for further development, including but not limited to improving ULF spatial resolution using DL tools trained on real‐world MRI data, we expect that solutions might have to be tailored to specific applications. Each application sets its own bar on *accuracy* and *precision*. Imaging protocols, reconstruction tools, or analysis approaches can be optimized for such properties, but ultimately, the balance between accuracy and precision must be aligned with the study objectives. One particular application of interest is the integration of ULF‐MRI data in normative brain charts to mitigate known demographic biases in study cohorts (Bethlehem et al. 2022). To achieve this goal, we must prioritize *accuracy* to obtain high agreement of MRI‐derived biomarkers across field strengths, regardless of age, sex, or clinical factors of interest, to promote harmonization. As more data is collected from clinical patients and healthy controls, or is pooled across studies, we will be able to test the impact of such factors on the outcome of comparative studies across field strengths. By doing so, we will learn to what extent ULF‐MRI provides an avenue to a more accessible counterpart of current neuroimaging techniques, and to what extent it encodes unique information on brain structure that must be better understood. This knowledge would promote the identification of applications for ULF‐MRI in clinical and research settings across disciplines.

## 5 Conclusions

ULF‐MRI is a promising technology for the quantitative analysis of brain structure. We observe strong correlations between brain volume features in subject‐matched ULF‐ and HF‐MRI data. However, some discrepancies can be attributed, in part, to the reduced spatial resolution of ULF‐MRI. We expect that new developments in DL technologies to enhance the spatial resolution and SNR will further promoting the utility of this more accessible and scalable neuroimaging technology. We further recommend the inclusion of coronally acquired data in imaging protocols to improve the accuracy and precision of brain volume analyses.

## Supporting information


**Data S1** Supporting Information.

## Data Availability

The data that support the findings of this study are available from the corresponding author upon reasonable request.
