# Supplementary material for: Morphological Brain Analysis Using Ultra Low‐Field MRI
Source: Hum Brain Mapp. 2025 Jun 30;46(10):e70232. doi: 10.1002/hbm.70232 (PMC12207323; doi:10.1002/hbm.70232)
Supplement: Supplementary file 1 — Data S1 Supporting Information. [file HBM-46-e70232-s001.docx]

**Supplementary Material**


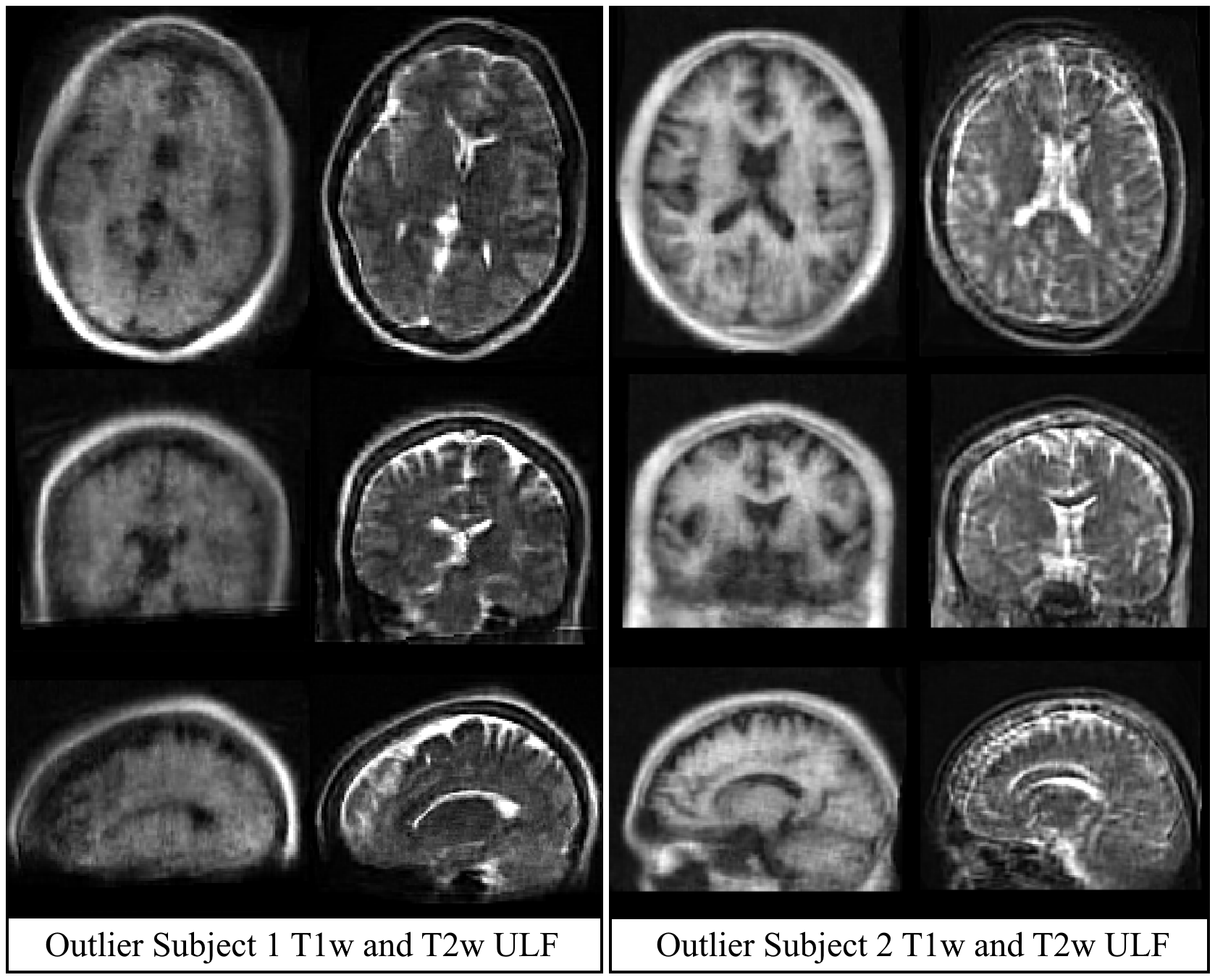


**Supplementary Figure 1.** The two identified outlier cases that were excluded from our ULF analysis. Outlier subject 1 had insufficient brain coverage on their T1w scan alongside excessive motion, resulting in an extremely blurry image. Outlier subject 2 had excessive motion between individual T2w ULF scans which could not be resolved through rigid registration or the ANTs multivariate template construction.


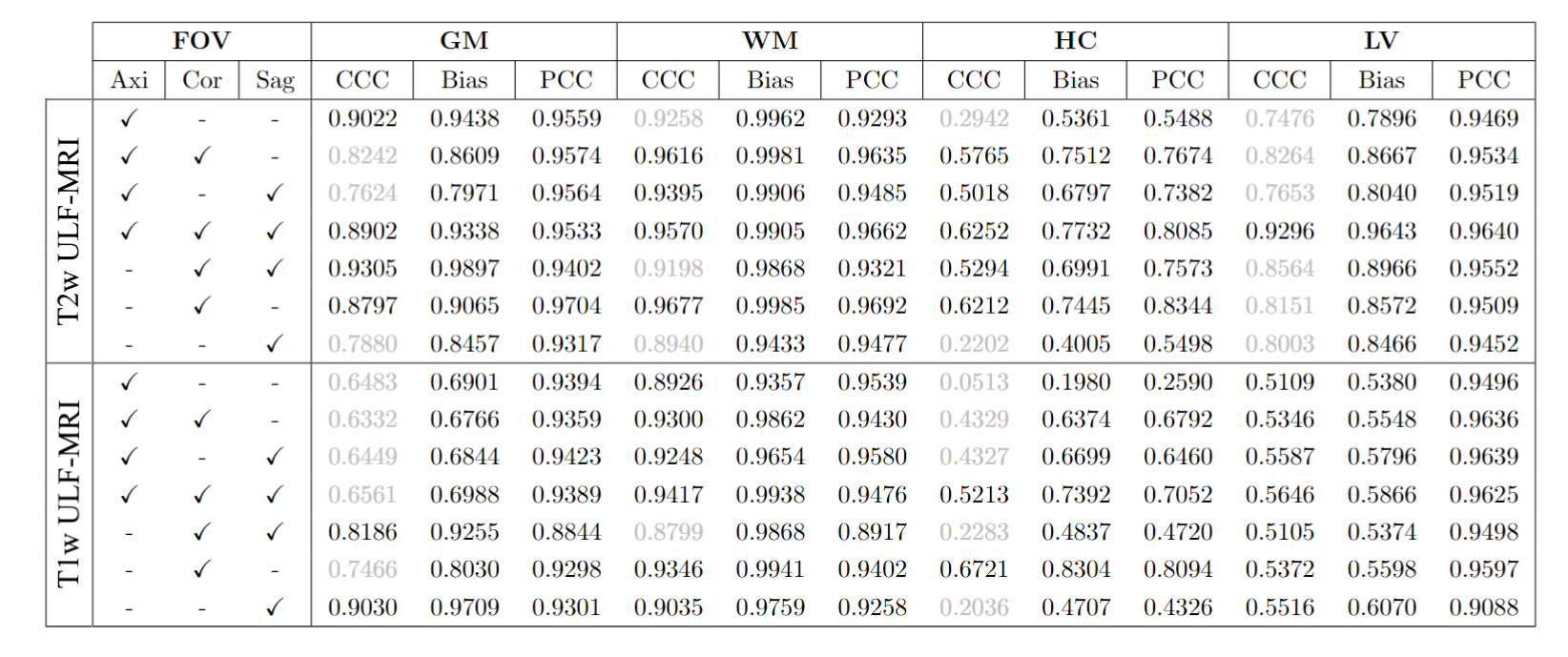


**Supplementary Table 1.** Full comparison between individual acquisition directions, partial combinations of two acquisition directions, and full combinations of three acquisition directions (TomoBrain), separately for T2w and T1w ULF-MRI. These evaluate the Lin’s CCC metric for clinical alignment with paired HF-MRI. CCC measurements that are significantly lower than the best performing results are colored with grayscale text to remove emphasis.


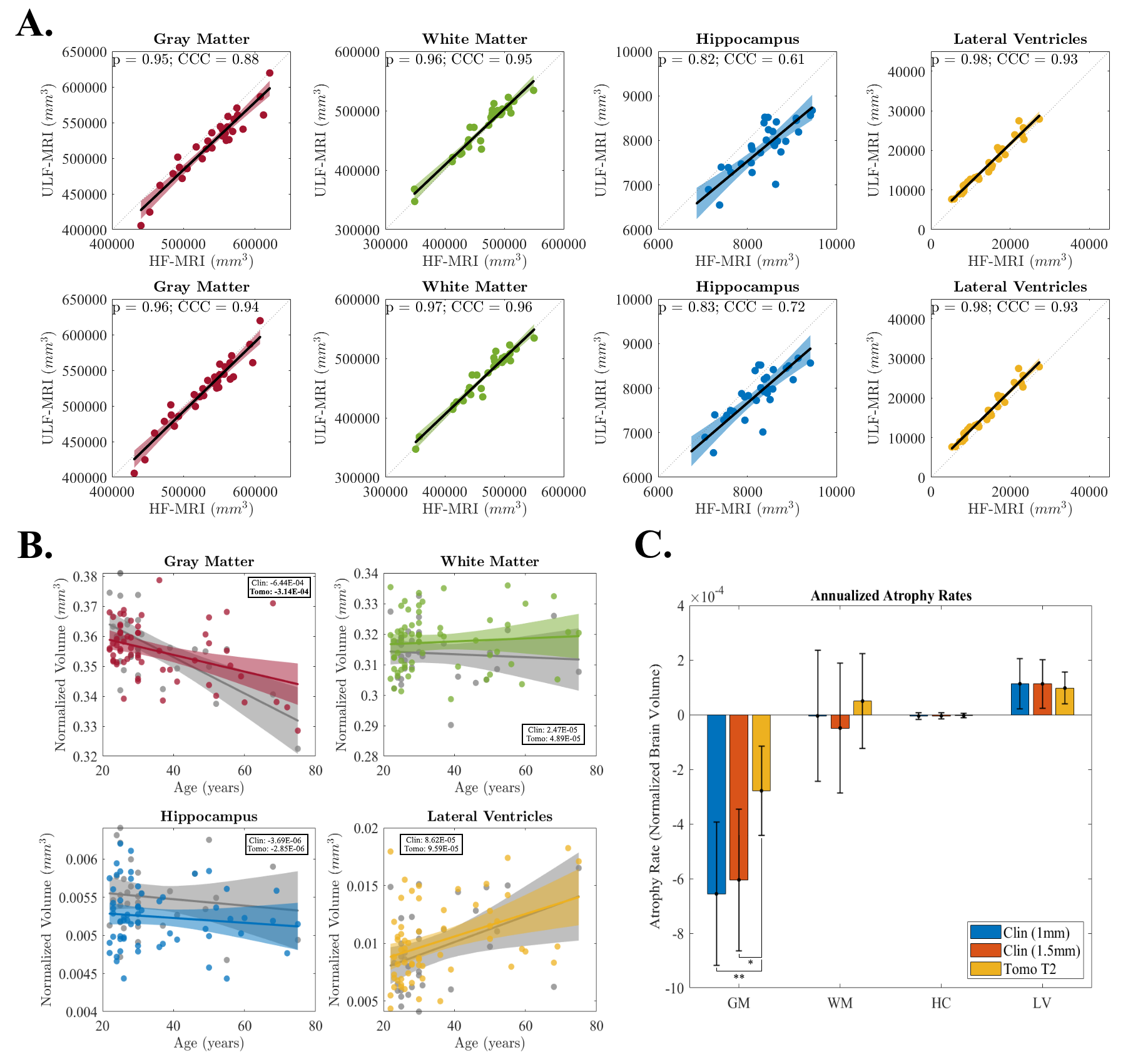


**Supplementary Figure 2. A.** Bivariate scatter plots of the brain volumes of interest for paired ULF-MRI (y-axis) and HF-MRI (x-axis). Row 1 showcases the comparison between T2w ULF TomoBrain at 1.5mm^3^ resolution and HF-MRI at 1mm^3^ resolution and Row 2 shows this for both T2w TomoBrain and HF-MRI at 1.5mm^3^ resolution. Pearson correlation coefficient and Lin’s CCC values for each comparison are shown in each plot. **B.** Age-volume trends for T2w TomoBrain (color) and HF-MRI (grayscale) at 1.5mm^3^ resolution for each brain volume of interest. Each brain region is normalized by ICV (y-axis) and plotted against age in years (x-axis). The trend lines are computed using linear regression with 95% confidence intervals. ULF trends that are significantly different from clinical trends are denoted with **bold**. **C.** Annualized atrophy rates computed for each volume of interest corresponding to the age-volume trends in **B.** with accompanying 95% confidence intervals. This is shown for our HF-MRI at 1mm^3^ (blue), our HF-MRI at 1.5mm^3^ (orange), and our T2w TomoBrain (yellow). Statistical significance at 95% and 99% confidence are denoted with * and **, respectively.
